# The IBR5 phosphatase promotes Arabidopsis auxin responses through a novel mechanism distinct from TIR1-mediated repressor degradation

**DOI:** 10.1186/1471-2229-8-41

**Published:** 2008-04-18

**Authors:** Lucia C Strader, Melanie Monroe-Augustus, Bonnie Bartel

**Affiliations:** 1 Department of Biochemistry and Cell Biology, Rice University, Houston, Texas 77005, USA

## Abstract

**Background:**

In Arabidopsis, INDOLE-3-BUTYRIC ACID RESPONSE5 (IBR5), a putative dual-specificity protein phosphatase, is a positive regulator of auxin response. Mutations in *IBR5 *result in decreased plant height, defective vascular development, increased leaf serration, fewer lateral roots, and resistance to the phytohormones auxin and abscisic acid. However, the pathways through which IBR5 influences auxin responses are not fully understood.

**Results:**

We analyzed double mutants of *ibr5 *with other mutants that dampen auxin responses and found that combining *ibr5 *with an auxin receptor mutant, *tir1*, enhanced auxin resistance relative to either parent. Like other auxin-response mutants, auxin-responsive reporter accumulation was reduced in *ibr5*. Unlike other auxin-resistant mutants, the Aux/IAA repressor reporter protein AXR3NT-GUS was not stabilized in *ibr5*. Similarly, the Aux/IAA repressor IAA28 was less abundant in *ibr5 *than in wild type. *ibr5 *defects were not fully rescued by overexpression of a mutant form of IBR5 lacking the catalytic cysteine residue.

**Conclusion:**

Our genetic and molecular evidence suggests that IBR5 is a phosphatase that promotes auxin responses, including auxin-inducible transcription, differently than the TIR1 auxin receptor and without destabilizing Aux/IAA repressor proteins. Our data are consistent with the possibility that auxin-responsive transcription can be modulated downstream of TIR1-mediated repressor degradation.

## Background

The phytohormone auxin is critical for plant growth and development, regulating vascular development, apical dominance, tropic responses, and organ patterning by modulating cell division and elongation [1,2]. Changes in gene expression are among the earliest molecular responses to auxin. Many auxin-responsive transcripts fall into one of three classes: *GH3*-related, *Auxin/INDOLE-3-ACETIC ACID *(*Aux/IAA*), and *SMALL AUXIN-UP RNA *(*SAUR*) transcripts [3-8]. Common to many of these auxin-responsive genes is a sequence in the upstream regulatory region termed the Auxin-Responsive Element (AuxRE; [9]).

AUXIN RESPONSE FACTOR (ARF) proteins are transcription factors that bind AuxREs (reviewed in [10]). Depending on the nature of the central domain, ARF family members can either activate or repress transcription [11,12]. ARF proteins can form homodimers, dimers with other ARF proteins, or dimers with transcriptionally repressive Aux/IAA proteins [13,14]. Many Aux/IAA proteins directly prevent transcriptional activation by interacting with activating ARF proteins [12,15].

Many Aux/IAA transcriptional repressors are unstable [5] and are degraded even more rapidly following auxin application [16,17]. Rapid Aux/IAA degradation following an auxin stimulus is thought to free activating ARF proteins from repression, allowing auxin-responsive gene expression. Mutant screens for decreased auxin sensitivity have identified several Aux/IAA proteins with stabilizing mutations (reviewed in [18]). Also isolated from auxin-response screens were trans-acting mutations that likewise stabilize Aux/IAA proteins, revealing the degradation mechanism for these repressors. Several auxin-resistant mutants have defects in the SCF^TIR1 ^E3 ubiquitin ligase complex, as well as its regulatory components (reviewed in [2]).

TRANSPORT INHIBITOR RESPONSE1 (TIR1) and the other AUXIN SIGNALING F-BOX (AFB) family members are the substrate-recognition components of SCF complexes that bind auxin and promote the degradation of Aux/IAA repressor proteins [19-21]. Auxin is trapped in the TIR1 auxin-binding pocket by an interacting Aux/IAA protein [22]. Subsequent 26S proteasomal degradation of Aux/IAA proteins relieves the repression of the ARF protein, allowing auxin-responsive transcription [16,17,23]. This novel receptor-ligand interaction allows a very short signal transduction chain that may facilitate rapid transcriptional responses to auxin. In addition, RUB (RELATED TO UBIQUITIN) modification of the CULLIN subunit of SCF^TIR1 ^is necessary for auxin response [24,25]. Mutations in *AXR1 *and *ECR1*, which encode subunits of the RUB-activating enzyme [26,27], result in decreased auxin responses accompanied by slowed Aux/IAA protein degradation [16,25,28-30], presumably because of reduced SCF^TIR1 ^efficacy in targeting these proteins for degradation.

Normal auxin responses require active movement of auxin through the plant, which is controlled by specialized influx and efflux carriers (reviewed in [31]). AUXIN RESISTANT1 (AUX1) is an auxin influx carrier protein that allows certain auxins to enter cells [32-35]. Mutations in *AUX1 *result in resistance to IAA and 2,4-dichlorophenoxyacetic acid (2,4-D) [34], which are substrates of the AUX1 transporter [35].

A variety of natural and synthetic auxins and auxin precursors have activity in auxin bioassays [2]. A mutation in *IBA RESPONSE5 *(*IBR5*) was identified in a screen for resistance to the inhibitory effects of the auxin precursor indole-3-butyric acid (IBA) on root growth [36]. Subsequent analyses revealed that *ibr5 *mutants are less sensitive not only to IBA, but also to all tested forms of auxin and to the phytohormone abscisic acid (ABA) [37]. *IBR5 *encodes a putative dual-specificity protein phosphatase, and the *ibr5-1 *mutation causes a premature stop codon that would result in a truncated product lacking the conserved phosphatase domain [37]. Here, we examined the role of IBR5 as a phosphatase *in vivo *by expressing a mutant version of IBR5 predicted to be catalytically inactive in the *ibr5 *mutant and found that phosphatase activity is likely required for full IBR5 function. Through double mutant analyses, we found that *ibr5 *enhanced most *tir1 *defects and a subset of *axr1 *and *aux1 *defects. Further, we demonstrated that *ibr5 *is defective in accumulation of an auxin-responsive reporter following auxin treatment. Because this reporter accumulates after degradation of Aux/IAA transcriptional repressors, we examined the effect of the *ibr5 *lesion on a reporter of Aux/IAA stability and an epitope-tagged Aux/IAA protein and, interestingly, found that these reporters were not stabilized in *ibr5*.

## Results

### *ibr5 *enhances *tir1 *auxin-response defects

**Figure 1:**
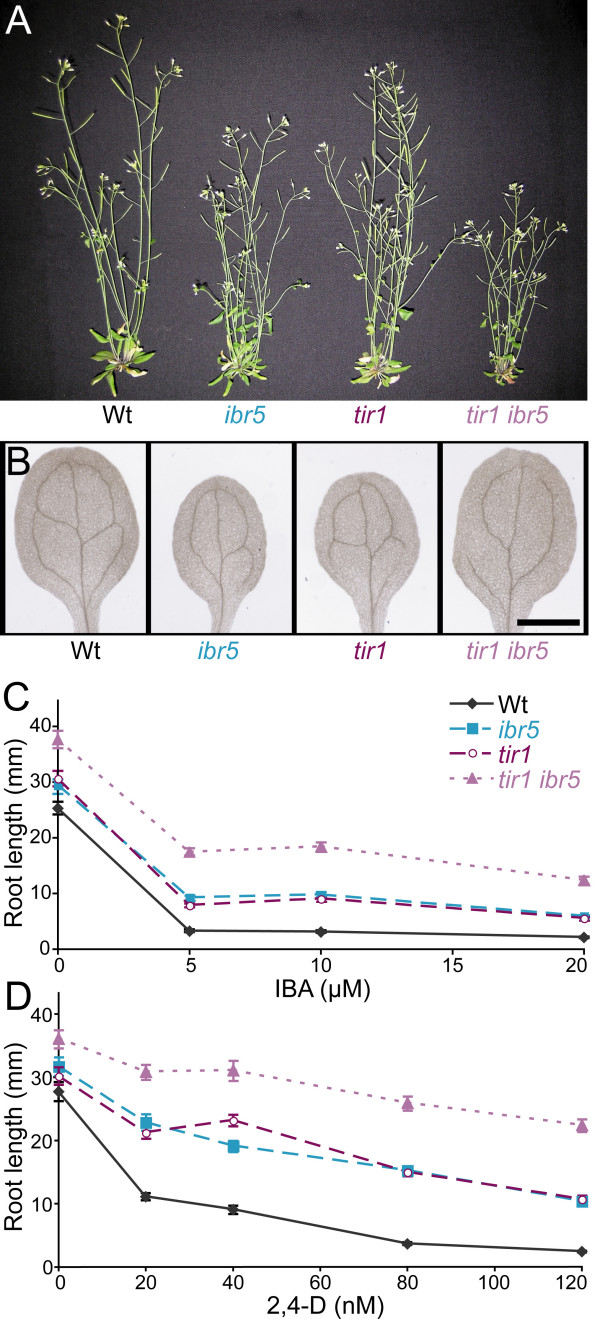
***tir1 ibr5 *morphological phenotypes and auxin response**. (A) Adult morphologies of wild-type, *ibr5*, *tir1*, and *tir1 ibr5 *plants. Six-week-old Col-0 (Wt), *ibr5-1*, *tir1-1*, and *tir1-1 ibr5-1 *grown in continuous light are shown. (B) Vascular patterning defects. Cleared cotyledons of 8-day-old Col-0 (Wt), *ibr5-1*, *tir1-1*, and *tir1-1 ibr5-1 *seedlings are shown. Scale bar = 1 mm. (C, D) *tir1-1 ibr5-1 *auxin-response defects. Lengths of primary roots of 8-day-old seedlings grown under yellow-filtered light at 22°C on medium supplemented with various concentrations of IBA (C) or 2,4-D (D) are shown. *tir1 ibr5 *roots were significantly longer than *tir1 *and *ibr5 *roots on control media and on all auxins tested (*P *≤ 0.001) in *t*-tests assuming unequal variance. Error bars represent standard errors of the means (*n *≥ 18).

**Figure 2:**
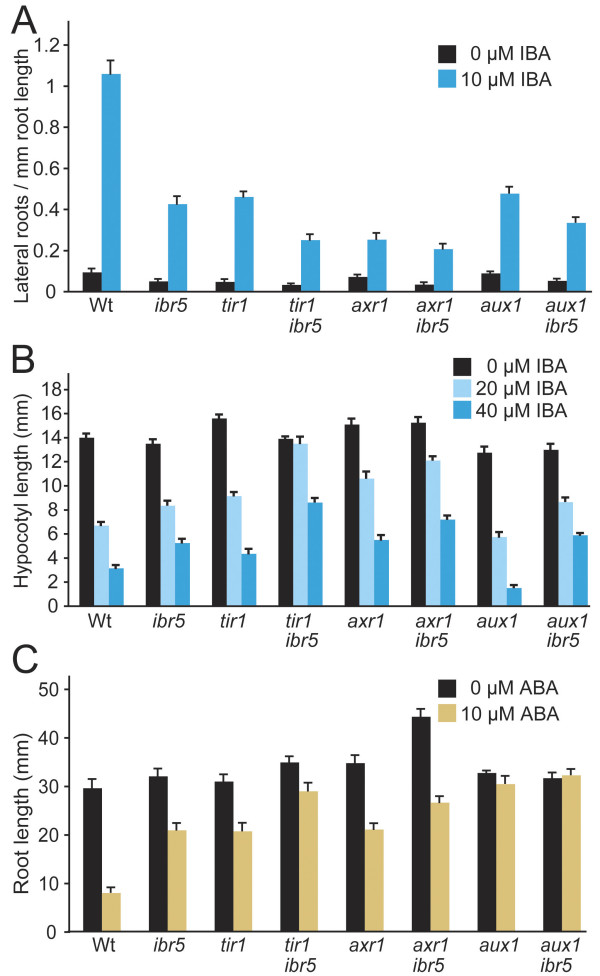
**Auxin-response mutant defects in lateral root induction by IBA, hypocotyl elongation inhibition by IBA, and root elongation inhibition by ABA**. Hormone response of Col-0 (Wt), *ibr5-1*, *tir1-1*, *tir1-1 ibr5-1*, *axr1-3*, *axr1-3 ibr5-1*, *aux1-7*, and *aux1-7 ibr5-1 *were examined. (A) Lateral roots were counted 4 days after transfer of 4-day-old seedlings to medium supplemented with either 0 (ethanol control) or 10 μM IBA. Primordia emerged from the main root were counted as lateral roots. Error bars represent standard errors of the means (*n *≥ 14). *tir1 ibr5 *had significantly fewer lateral roots in response to IBA than either *tir1 *or *ibr5 *(*P *≤ 0.001 in two-tailed *t*-tests assuming unequal variance). (B) Lengths of hypocotyls were measured 4 days after transfer of 1-day-old seedlings to the dark. Error bars represent standard errors of the means (*n *= 20). *tir1 ibr5 *hypocotyls were significantly longer than *tir1 *and *ibr5 *hypocotyls on 20 or 40 μM IBA (*P *≤ 0.0001 in two-tailed *t*-tests assuming unequal variance). *axr1 ibr5 *hypocotyls were significantly longer than *axr1 *and *ibr5 *on 20 (*P *≤ 0.01) or 40 μM IBA (*P *≤ 0.001) in two-tailed *t*-tests assuming unequal variance. (C) Length of primary roots 4 days after transfer of 4-day-old seedlings to medium supplemented with either 0 (ethanol control) or 10 μM ABA. *tir1 ibr5 *roots were significantly longer than *tir1 *and *ibr5 *roots on ABA (*P *≤ 0.001) in two-tailed *t*-tests assuming unequal variance. *axr1 ibr5 *roots were significantly longer than *axr1 *and *ibr5 *roots following control (*P *≤ 0.001) or ABA (*P *≤ 0.01) treatments in two-tailed *t*-tests assuming unequal variance. Error bars represent standard errors of the means (*n *≥ 14).

The *tir1 *mutant, like *ibr5 *[37], is less responsive to auxin in primary root elongation inhibition and lateral root formation assays [38]. To examine the genetic interaction between *ibr5 *and *tir1*, we crossed *tir1-1 *to *ibr5-1 *and examined the phenotypes of the resulting double mutant. We found that *tir1 ibr5 *plants were shorter than either parent (Figure 1A). *ibr5 *cotyledon vascularization defects were sometimes mildly enhanced by *tir1 *(Figure 1B). In addition, the *tir1 ibr5 *double mutant displayed enhanced resistance to root elongation inhibition by 2,4-D and IBA (Figures 1C and 1D, Additional File 1), fewer lateral roots in response to IBA treatment (Figure 2A), and greater resistance to IBA inhibition of hypocotyl elongation in the dark (Figure 2B, Additional File 2) than either parent.

In addition to auxin resistance, *ibr5 *mutant roots are resistant to the phytohormone ABA [37]. We found that *tir1 *also exhibited ABA resistance, and that *tir1 ibr5 *roots were more ABA resistant than either single mutant (Figure 2C, Additional File 2). Because *ibr5-1 *is likely to be a null allele [37], these results support a model in which IBR5 and TIR1 act separately to affect auxin and ABA responsiveness.

### *ibr5 *enhances certain *axr1 *auxin-response defects

**Figure 3:**
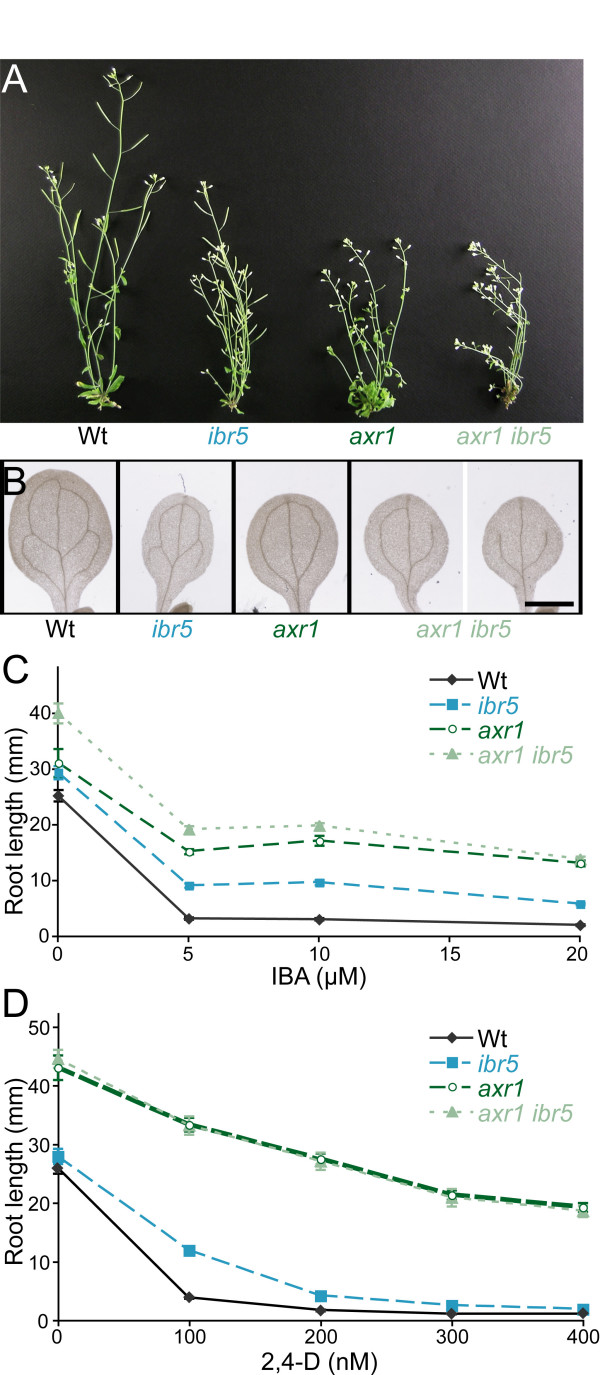
***axr1 ibr5 *morphological phenotypes and auxin response**. (A) Adult morphologies of wild-type, *ibr5*, *axr1*, and *axr1 ibr5 *plants. Six-week-old Col-0 (Wt), *ibr5-1*, *axr1-3*, and *axr1-3 ibr5-1 *grown in continuous light are shown. (B) Vascular patterning defects. Cleared cotyledons of 8-day-old Col-0 (Wt), *ibr5-1*, *axr1-3*, and *axr1-3 ibr5-1 *seedlings are shown. Scale bar = 1 mm. (C, D) *axr1-3 ibr5-1 *auxin-response defects. Lengths of primary roots of 8-day-old (C) or 9-day-old (D) seedlings grown under yellow-filtered light at 22°C on medium supplemented with various concentrations of IBA (C) or 2,4-D (D) are shown. *axr1 ibr5 *roots were significantly longer than *axr1 *and *ibr5 *on 0 (*P *≤ 0.01), 5 (*P *≤ 0.001), and 10 (*P *≤ 0.001) μM IBA in two-tailed *t*-tests assuming unequal variance. *axr1 ibr5 *roots were not significantly different from *axr1 *roots on tested 2,4-D concentrations. Error bars represent standard errors of the means (*n *≥ 15).

The *axr1 *mutant displays more extreme auxin-response defects than *tir1 *or *ibr5*, with restricted plant height, reduced apical dominance, dramatic vascularization defects, striking auxin resistance, and a longer root than wild type on unsupplemented media [29]. To examine the genetic interaction between *axr1 *and *ibr5*, we crossed *axr1-3 *to *ibr5-1*. The double mutant had similar plant height to *axr1-3 *(Figure 3A), but leaf epinasty (data not shown) and cotyledon vascular defects (Figure 3B) were more extreme in *axr1 ibr5 *compared to either parent. Further, *axr1 ibr5 *had a longer root on unsupplemented media than either parent (Figure 3C), consistent with the possibility that resistance to endogenous auxin was enhanced. Resistance to the auxins 2,4-D and IBA was not obviously enhanced in the double mutant when considering the longer root on unsupplemented media (Figures 3C and 3D, Additional File 1). Moreover, *axr1 ibr5 *did not display enhanced resistance to IBA-induced lateral root formation (Figure 2A, Additional File 2), but did exhibit slightly enhanced resistance to the inhibition by IBA of hypocotyl elongation in the dark (Figure 2B, Additional File 2). Like *ibr5*, *axr1 *is resistant to ABA inhibition of root elongation [37], and *axr1 ibr5 *had similar ABA resistance as both parents (Figure 2C, Additional File 2).

### *ibr5 *enhances *aux1 *root elongation defects

**Figure 4:**
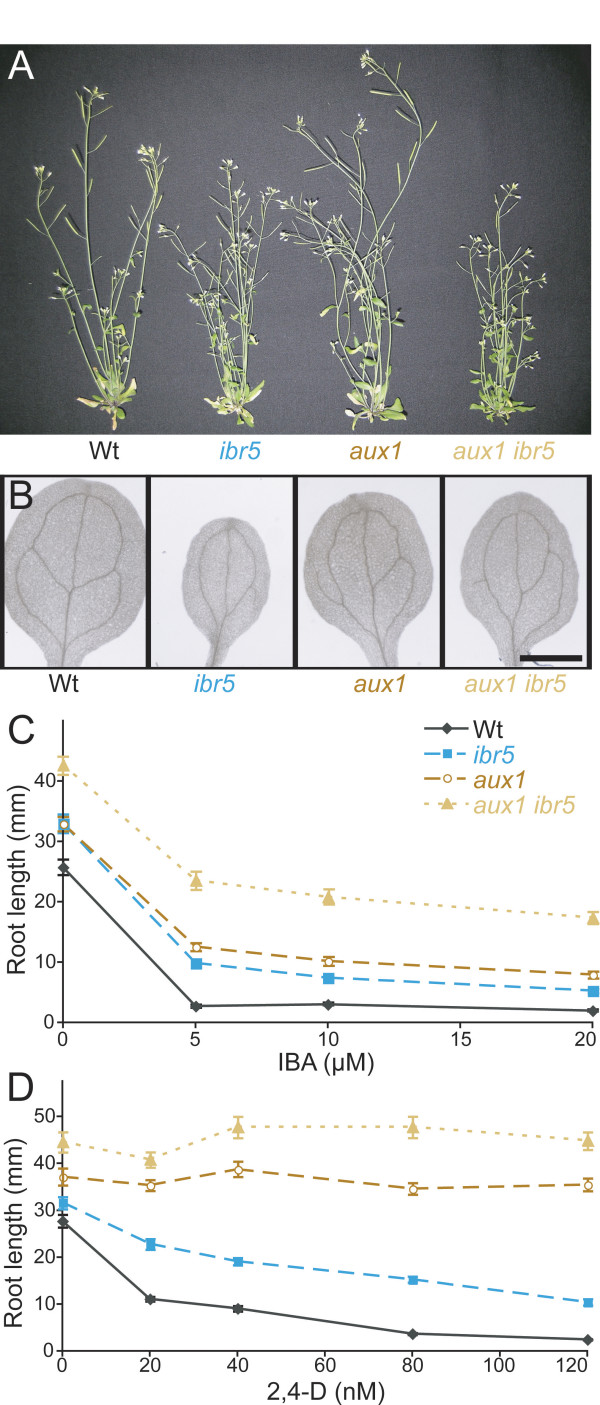
***aux1 ibr5 *morphological phenotypes and auxin response**. (A) Adult morphologies of wild-type, *ibr5*, *aux1*, and *aux1 ibr5 *plants. Six-week-old Col-0 (Wt), *ibr5-1*, *aux1-7*, and *aux1-7 ibr5-1 *grown in continuous light are shown. (B) Vascular patterning defects. Cleared cotyledons of 8-day-old Col-0 (Wt), *ibr5-1*, *aux1-7*, and *aux1-7 ibr5-1 *seedlings are shown. Scale bar = 1 mm. (C, D) *aux1-7 ibr5-1 *auxin-response defects. Lengths of primary roots of 8-day-old seedlings grown under yellow-filtered light at 22°C on medium supplemented with various concentrations of IBA (C) or 2,4-D (D) are shown. *aux1 ibr5 *roots were significantly longer than *aux1 *and *ibr5 *roots in the absence of hormone and on 5, 10, and 20 μM IBA (*P *≤ 0.001) in two-tailed *t*-tests assuming unequal variance. *aux1 ibr5 *roots were significantly longer than *aux1 *and *ibr5 *roots on 20 (*P *≤ 0.01), 40 (*P *≤ 0.01), 80 (*P *≤ 0.0001), and 120 (*P *≤ 0.001) nM 2,4-D in two-tailed *t*-tests assuming unequal variance. Error bars represent standard errors of the means (*n *≥ 16).

The *aux1 *mutant displays marked resistance to the auxins that are brought into cells by the AUX1 transporter, such as 2,4-D and IAA [33,34,39], but responds normally to 1-naphthaleneacetic acid (NAA), which is not transported by AUX1 [33-35]. Although IBA does not appear to be an AUX1 substrate [35,40], the *aux1 *mutant is moderately IBA resistant [36], probably because the IBA that enters cells is converted to IAA. *aux1 *mutant roots are agravitropic and longer than wild-type roots on unsupplemented media [39], but *aux1 *aerial parts resemble wild type. To examine the genetic interaction between *aux1 *and *ibr5*, we crossed *ibr5-1 *to *aux1-7*. Although *aux1 *plants attain normal height, adult *aux1 ibr5 *plants were shorter than either parent (Figure 4A). Moreover, *aux1 ibr5 *seedlings had longer roots on unsupplemented media than either parent (Figure 4C). Resistance to the auxins 2,4-D and IBA was not obviously enhanced in the double mutant when considering the longer root on unsupplemented media and the complete resistance of *aux1 *to the concentrations of 2,4-D tested (Figures 4C and 4D, Additional File 1). We examined lateral root production in *aux1 ibr5 *and found that *aux1 *did not markedly enhance *ibr5 *defects (Figure 2A, Additional File 2). Similarly, the *ibr5 *cotyledon vascular development defects did not appear to be enhanced by *aux1 *(Figure 4B). Hypocotyls of dark-grown *aux1 *responded like wild type to IBA, and *aux1 ibr5 *responses resembled those of *ibr5 *(Figure 2B, Additional File 2). *aux1 *was unresponsive to the ABA concentrations tested (Figure 2C, Additional File 2); thus we did not determine if *ibr5 *enhanced *aux1 *ABA resistance.

### *ibr5 *displays reduced auxin-responsive reporter accumulation

**Figure 5:**
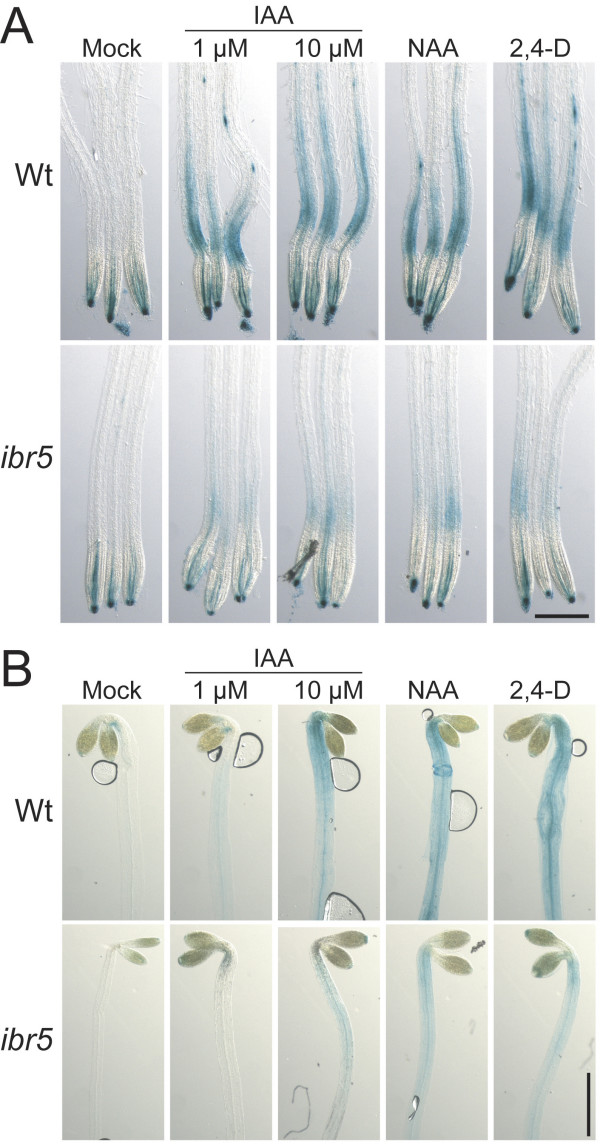
**An auxin-responsive reporter is reduced in *ibr5***. (A) 8-day-old light-grown Col-0 (Wt) and *ibr5-1 *seedlings carrying the *DR5:GUS *construct [13, 37] were mock treated or treated with 1 μM IAA, 10 μM IAA, 10 μM NAA, or 10 μM 2,4-D for 2 hours, then stained for GUS activity. Scale bar = 0.5 mm. (B) 5-day-old dark-grown Col-0 (Wt) and *ibr5-1 *seedlings carrying the *DR5:GUS *construct [13, 37] were mock treated or treated with 1 μM IAA, 10 μM IAA, 10 μM NAA, or 10 μM 2,4-D for 2 hours, then stained for GUS activity. Scale bar = 1 mm.

*ibr5 *seedlings grown on unsupplemented medium display reduced accumulation of *DR5:GUS *[37], a construct in which the GUS reporter is driven from a synthetic auxin-responsive promoter [13]. We compared wild-type and *ibr5 *DR5:GUS auxin responses in roots of light-grown seedlings and hypocotyls of dark-grown seedlings. Two-hour treatments with various auxins increased DR5:GUS activity in wild-type roots (Figure 5A) and hypocotyls (Figure 5B). In contrast, *ibr5 *showed reduced induction of DR5:GUS activity following auxin treatment in both roots and hypocotyls (Figures 5A and 5B).

This reduced induction of DR5:GUS activity suggests that *ibr5 *misregulates at least some auxin-regulated transcripts. Thus, we examined basal levels and auxin responsiveness of endogenous *IAA1*, *IAA2*, and *GH3.3 *transcripts in wild-type and *ibr5 *seedlings. Although we detected subtle reductions in basal levels of *IAA1 *and *IAA2 *transcripts in *ibr5 *in several trials (data not shown), we did not detect dramatic differences in these experiments. In any case, *IAA1*, *IAA2*, and *GH3.3 *transcripts eventually reached similar maximal values in wild type and *ibr5 *(data not shown), suggesting that IBR5 is not required for full response to high auxin levels.

### *ibr5 *does not accumulate an AXR3/IAA17 reporter protein

Gain-of-function mutations that stabilize any of several Aux/IAA proteins can confer dominant auxin resistance (reviewed in [18]). Moreover, Aux/IAA repressor proteins or Aux/IAA-reporter fusion proteins are stabilized in numerous other auxin-resistant mutants, including *tir1 *[16], *axr1 *[16], *ecr1 *[28], *afb1*, *afb2*, and *afb3 *[21], *cul1 *[41], *eta2/cand1 *[42], *eta3/sgt1b *[43], and *aar1 *[44].

**Figure 6:**
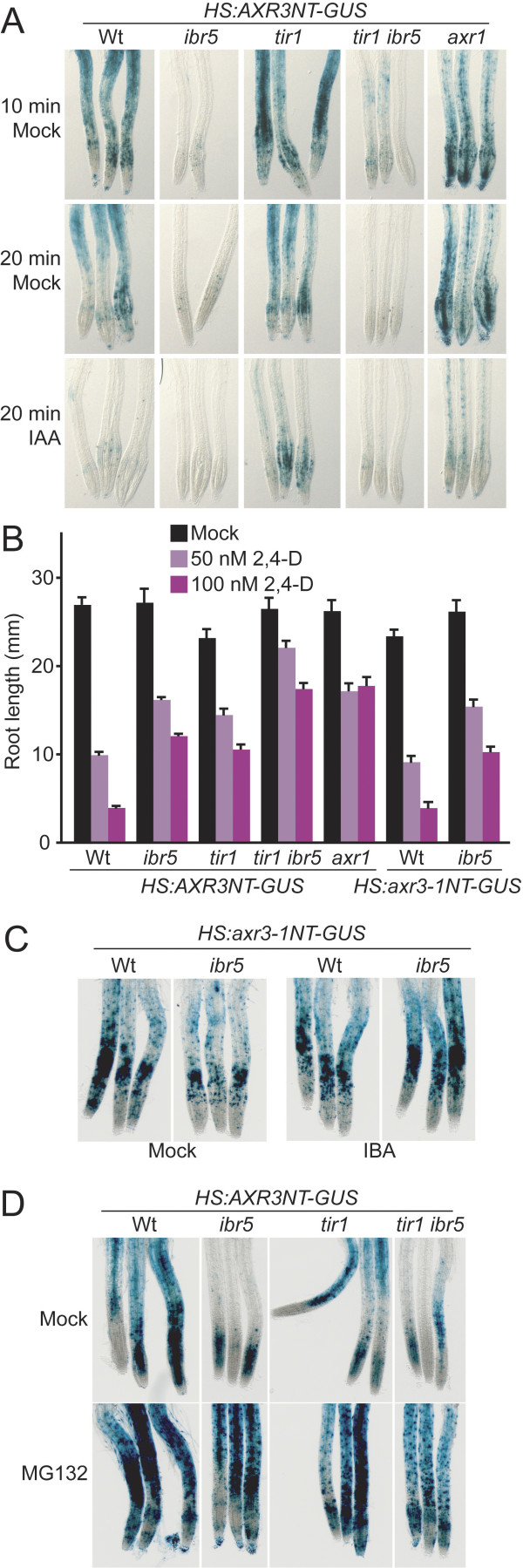
***ibr5 *does not accumulate AXR3NT-GUS**. (A) 8-day-old Col-0 (Wt), *ibr5-1*, *tir1-1*, *tir1-1 ibr5-1*, and *axr1-3 *seedlings carrying *HS:AXR3NT-GUS *[16] were heat shocked for 2 hours, treated with mock (ethanol) or 100 nM IAA for the indicated time, then stained for GUS activity. (B) Auxin-response defects of *HS:AXR3NT-GUS *lines. Lengths of primary roots of 8-day-old seedlings grown under yellow-filtered light at 22°C on medium supplemented with various concentrations of 2,4-D are shown. Error bars represent standard errors of the means (*n *= 20). (C) 8-day-old Col-0 (Wt) and *ibr5-1 *carrying *HS:axr3-1NT-GUS *[16] were heat-shocked for 2 hours, mock (ethanol) treated or treated with 10 μM IBA for 40 minutes, then stained for GUS activity. (D) 8-day-old Col-0 (Wt), *ibr5-1*, *tir1-1*, and *tir1-1 ibr5-1 *carrying *HS:AXR3NT-GUS *[16] were heat shocked for 2 hours. Midway through a 2-hour heat shock, DMSO (mock) or 50 μM MG132 treatment was initiated. Seedlings were stained for GUS activity 2 hours after return to room temperature. Separate experiments revealed that inclusion of DMSO during the heat shock (included as an MG132 carrier in panel D) resulted in more intense AXR3NT-GUS staining (L.C.S., unpublished), which could account for the higher apparent GUS activity in panel D when compared to panel A.

Because auxin-responsive transcripts are reduced in *ibr5*, we sought to analyze Aux/IAA stability in the *ibr5 *mutant. We crossed *ibr5-1 *to a line expressing the AXR3/IAA17 Aux/IAA protein N-terminal degron region fused to β-glucuronidase driven by a soybean heat-shock promoter (*HS:AXR3NT-GUS*; [16]). Eight-day-old seedlings were heat shocked to induce reporter transcription, and then either mock treated or auxin treated. In seedlings with intact auxin signaling, the auxin-induced disappearance of AXR3NT-GUS activity reflects the targeting of the reporter to the 26S proteasome for degradation [16]. Mutants with auxin-response defects, such as *tir1 *and *axr1*, show increased reporter activity after induction and reduced destabilization of AXR3NT-GUS upon auxin treatment [16], consistent with the *axr1 *defect in accumulating normal levels of auxin-responsive transcripts following auxin treatment [45,46]. Intriguingly, unlike in previously characterized auxin-response mutants, we did not detect increased AXR3NT-GUS activity in *ibr5*. In fact, AXR3NT-GUS appeared to be less active in *ibr5 *than in wild-type roots 10 or 20 minutes following heat shock (Figure 6A). This decrease in AXR3NT-GUS activity was also apparent in *tir1 ibr5*, suggesting that *ibr5*, although enhancing *tir1 *auxin resistance in root elongation (Figures 1C, D, and 6B), suppressed *tir1 *AXR3NT-GUS accumulation (Figure 6A). Response to 2,4-D for each of these lines was as expected, with *tir1 ibr5 *(*HS:AXR3NT-GUS*) showing enhanced resistance compared to the intermediate resistance of either parent (Figure 6B).

The reduced AXR3NT-GUS activity in *ibr5 *was apparent immediately following the 2-hour heat shock used to induce reporter expression (data not shown). Because a heat-responsive promoter drives the AXR3NT-GUS construct, we tested whether the lack of AXR3NT-GUS activity in *ibr5 *could be explained by a reduced transcriptional response to heat in the mutant. We introduced a construct altered to contain the stabilizing axr3-1 mutation (*HS:axr3-1NT-GUS*; [16]) into *ibr5-1 *by crossing. The proline to leucine substitution in axr3-1 [47] confers reporter stability by decreasing axr3-1NT-GUS interaction with SCF^TIR1 ^[16]. We found similar axr3-1NT-GUS activity in wild type and *ibr5 *with or without auxin treatment (Figure 6C), suggesting that the transgenes were efficiently transcribed in response to the heat stimulus, and that the decreased AXR3NT-GUS activity in *ibr5 *was not caused by reduced transcription following heat shock. Moreover, treatment with the proteasome inhibitor MG132 restored AXR3NT-GUS activity in *ibr5 *and *tir1 ibr5 *to near wild-type levels (Figure 6D), again suggesting that the *ibr5 *defects in AXR3NT-GUS activity were not due to reduced transgene transcription.

### *ibr5 *does not accumulate an IAA28 reporter protein

**Figure 7:**
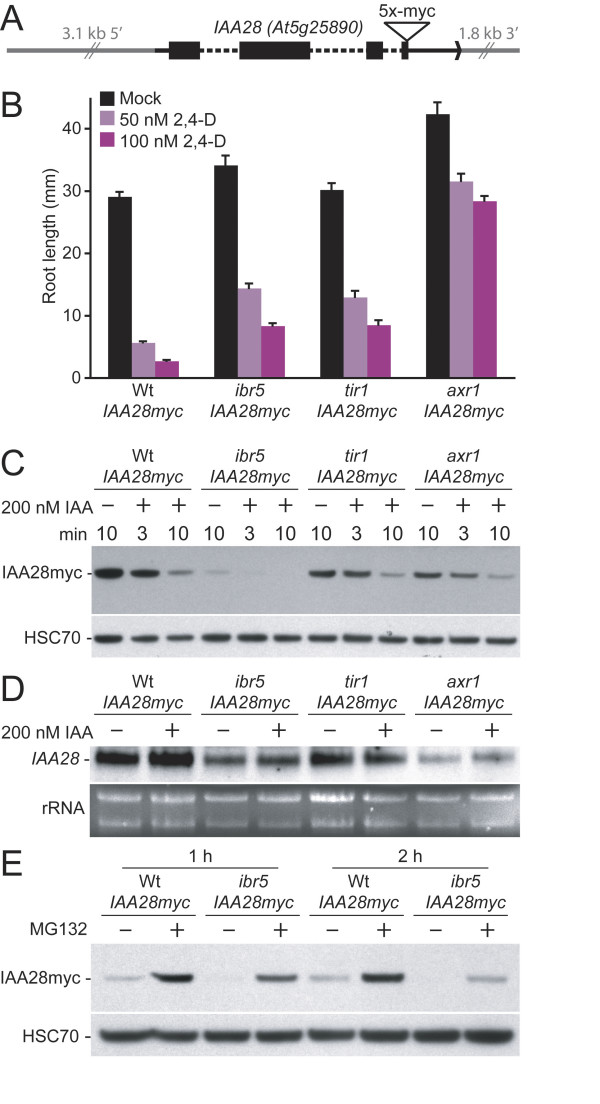
***ibr5 *does not accumulate IAA28myc**. (A) An illustration of the *IAA28myc *construct. The region of the *IAA28 *transcript is shown in black, with introns designated with dashed lines and coding sequence with black boxes. (B) Auxin-response defects of indicated mutants carrying the *IAA28myc *construct. Lengths of primary roots of 8-day-old seedlings grown under yellow-filtered light at 22°C on medium supplemented with the indicated concentrations of 2,4-D are shown. Error bars represent standard errors of the means (*n *= 17). (C) IAA28myc accumulation in wild type and auxin-response mutants. Anti-myc (top panel; Santa Cruz Biotechnology) and anti-HSC70 antibodies (bottom panel; Stressgen Bioreagents) were used on immunoblots of protein prepared from roots of light-grown 10-day-old Col-0 (Wt), *ibr5-1*, *tir1-1*, and *axr1-3 *seedlings expressing IAA28myc that had been mock (ethanol) treated for 10 minutes or treated for 3 or 10 minutes with 200 nM IAA. (D) *IAA28 *mRNA accumulation in wild type and auxin response mutants. Total RNA from seedlings that had been mock (ethanol) treated or treated with 200 nM IAA for 10 minutes was separated by electrophoresis (bottom panel, ethidium bromide-stained gel), transferred to a membrane, and probed with an *IAA28 *probe (top panel). *IAA28 *and *IAA28myc *transcripts are not resolved from one another, and therefore are seen as a single band. (E) IAA28myc accumulation in response to MG132 treatment. Anti-myc (top panel) and anti-HSC70 (bottom panel) antibodies were used on immunoblots of protein prepared from 3-day-old light-grown Col-0 (Wt) and *ibr5-1 *seedlings expressing IAA28myc that had been mock (DMSO) treated or treated with 300 μM MG132 for 1 or 2 hours.

*IAA28 *was originally identified because the *iaa28-1 *gain-of-function mutation confers auxin resistance and impedes lateral root production [48]. Like typical Aux/IAA proteins, IAA28 has a transcriptional repressor domain [15] and can confer auxin-enhanced instability to a luciferase reporter [49]. To test whether the lack of AXR3NT-GUS stabilization in *ibr5 *was accompanied by stability effects on other Aux/IAA proteins, we crossed *ibr5-1*, *tir1-1*, and *axr1-3 *to a wild-type line carrying a c-Myc epitope-tagged version of IAA28 driven from *IAA28 *regulatory sequences (Figure 7A) and isolated homozygous mutants carrying the reporter transgene. These lines responded to 2,4-D as expected (Figure 7B). Because IAA28 is primarily expressed in roots [48], we examined accumulation of IAA28myc in wild-type and mutant roots following mock- or auxin-treatment of 10-day-old seedlings. As expected, we found that IAA28myc disappeared rapidly following auxin treatment in wild type (Figure 7C). We found lower IAA28myc levels in *ibr5 *than wild type in the absence of added auxin; this protein disappeared rapidly upon auxin treatment and was not detected after 10 minutes of treatment (Figure 7C). Although we had expected IAA28myc to be stabilized in *tir1 *and *axr1*, we found IAA28myc levels similar to wild-type levels that decreased in response to auxin treatment (Figure 7C) in both mutants, consistent with the observation that neither of these mutants is completely insensitive to auxin (e.g., Figure 7B). We examined *IAA28 *mRNA levels in these lines and found reduced *IAA28 *transcript levels in *axr1 *and, to a lesser extent, in *tir1 *(Figure 7D). Transcript levels were not perfectly correlated with IAA28 protein levels, suggesting differences in protein stability in these mutants. Treatment with MG132 increased IAA28myc protein levels in both wild type and *ibr5 *(Figure 7E), suggesting that IAA28myc is degraded via the 26S proteasome in wild type and consistent with the possibility that the proteasome contributes to the reduced IAA28myc levels in *ibr5*. However, the inability of MG132 to fully restore IAA28myc to wild-type levels in *ibr5 *(Figure 7E) suggests that the reduced *IAA28 *mRNA level in *ibr5 *(Figure 7D) contributes to the reduced IAA28myc accumulation in this mutant (Figure 7C). The striking lack of AXR3NT-GUS and IAA28myc stabilization in *ibr5 *is consistent with the possibility that auxin-regulated transcription is reduced in *ibr5 *via a mechanism that does not involve Aux/IAA protein stabilization.

### An IBR5 substitution variant (IBR5^C129S^) does not fully rescue *ibr5 *defects

Dual specificity protein phosphatase (DSP) proteins dephosphorylate both threonine and tyrosine residues of phosphorylated proteins, often thereby inactivating them (reviewed in [50]). DSP proteins contain a conserved aspartate residue and a separate, highly conserved signature motif of VxVHCx_2_GxSRSx_5_AYLM, with the cysteine and arginine residues participating with the conserved aspartate in catalysis. The cysteine of this signature begins the dephosphorylation process with a nucleophilic attack on the phosphorus atom of the phosphotyrosine or phosphothreonine substrate. Thus, disruption of this conserved cysteine results in catalytic inactivity in many DSP proteins (reviewed in [51]), including the IBR5 relative, DsPTP1 [52].

**Figure 8:**
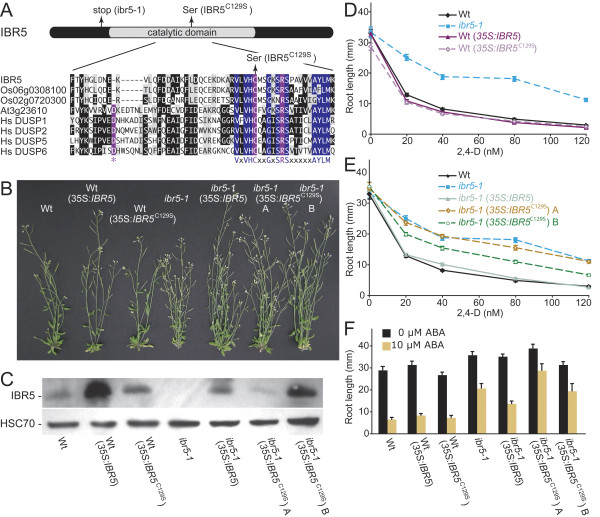
**An IBR5^C129S ^substitution variant does not fully rescue *ibr5 *defects**. (A) A schematic showing the positions of the ibr5-1 premature stop codon relative to the conserved catalytic domain and an alignment of part of the phosphatase catalytic domains of Arabidopsis IBR5 and several putative and confirmed DSP proteins. Sequences shown are the closest IBR5 homologs from rice (Os06g0308100 and Os02g0720300), an IBR5 relative from Arabidopsis with demonstrated DSP activity (At3g23610/DsPTP1; [52]) and three human (Hs) DSP enzymes. Sequences were aligned with the MegAlign program (DNAStar, Madison, WI) using the CLUSTAL W method. Catalytic residues are shaded in purple, conserved DSP signature residues are shaded in blue, residues identical in at least four sequences are shaded in black, similar residues are shaded in gray, and dashes indicate gaps introduced to maximize alignment. (B) Six-week-old Col-0 (Wt), Wt (*35S:IBR5*), Wt (*35S:IBR5*^*C*129*S*^), *ibr5-1*, *ibr5-1 *(*35S:IBR5*), *ibr5-1 *(*35S:IBR5*^*C*129*S*^) line A, and *ibr5-1 *(*35S:IBR5*^*C*129*S*^) line B grown in continuous light are shown. (C) Immunoblot analysis with an anti-IBR5 antibody [37]; top panel) and anti-HSC70 antibody (Stressgen Bioreagents) of protein prepared from 2-day-old seedlings of the lines shown in panel B. Positions of IBR5 and HSC70 are indicated at left. (D) Wt (*35S:IBR5*) and Wt (*35S:IBR5*^*C*129*S*^) display similar 2,4-D response as Wt. Lengths of primary roots of 8-day-old seedlings grown under yellow-filtered light at 22°C on medium supplemented with various concentrations of 2,4-D are shown. Error bars represent standard errors of the means (*n *≥ 18). (E) *ibr5-1 *(*35S:IBR5*^*C*129*S*^) line A, and *ibr5-1 *(*35S:IBR5*^*C*129*S*^) line B fail to fully rescue *ibr5-1 *2,4-D resistance. Seedlings were measured as in (D). Error bars represent standard errors of the means (*n *≥ 17). (F) Length of primary roots 4 days after transfer of 4-day-old seedlings to either 0 (ethanol control) or 10 μM ABA medium. Error bars represent standard errors of the means (*n *≥ 8).

The DSP active site motif VxVHCx_2_GxSRSx_5_AYLM is present in IBR5 (Figure 8A), allowing us to identify the presumptive active site cysteine (C129) in IBR5. To test whether IBR5 phosphatase activity is required for normal auxin responses *in vivo*, we generated transgenic Wt and *ibr5-1 *lines expressing a Cys129 → Ser129 (C129S) substitution variant of IBR5 (IBR5^C129S^) under the control of the strong 35S viral promoter. We anticipated that *35S:IBR5*^*C*129*S *^would not rescue *ibr5-1 *defects if IBR5 phosphatase activity were required to promote auxin responsiveness. We assayed *ibr5-1 *(*35S:IBR5*^*C*129*S*^) lines expressing low (line A) and high (line B) IBR5^C129S ^levels (Figure 10C) for mutant phenotype rescue. Wild-type and *ibr5-1 *lines overexpressing unmodified IBR5 (*35S:IBR5*; [37]) were used for comparison.

We found that IBR5 or IBR5^C129S ^overexpression did not noticeably alter wild-type adult morphology (Figure 8B). Moreover, IBR5 or IBR5^C129S ^overexpression in wild type did not alter any of the hormone-response phenotypes examined (Figure 8D, F). As expected, we found that *ibr5-1 *(*35S:IBR5*) plants were restored to wild-type height (Figure 8B) and had normal root responses to auxin and ABA (Figure 8E, F). *ibr5-1 *(*35S:IBR5*^*C*129*S*^) line A (low expressor) exhibited restored plant height but had similar leaf epinasty to *ibr5-1*, whereas *ibr5-1 *(*35S:IBR5*^*C*129*S*^) line B (high expressor) displayed rescue of *ibr5-1 *plant height (Figure 8B) and partial rescue of leaf epinasty (data not shown). Resistance to the inhibitory effects of 2,4-D and ABA on root elongation was not rescued in *ibr5-1 *(*35S:IBR5*^*C*129*S*^) line A and only partially restored in *ibr5-1 *(*35S:IBR5*^*C*129*S*^) line B (Figures 8E, F). The lack of full *ibr5-1 *rescue by IBR5^*C*129*S *^is consistent with the possibility that IBR5 phosphatase activity is required for full auxin and ABA responsiveness. The partial *ibr5-1 *rescue observed when *IBR5*^*C*129*S *^accumulates to high levels suggests that certain IBR5 functions do not require phosphatase activity. For example, IBR5^C129S ^may bind and sequester its substrate(s), thereby dampening normal substrate activity.

## Discussion

Loss-of-function mutations in *IBR5*, which encodes a putative dual-specificity protein phosphatase (DSP), result in decreased auxin and abscisic acid responses [37]. DSP proteins often regulate mitogen-activated protein kinase (MAPK) proteins. Arabidopsis has 20 predicted MAPK proteins [53] but only five predicted DSP proteins, suggesting that some DSP enzymes may regulate more than one MAPK. In Arabidopsis, two of the five predicted Arabidopsis DSP proteins have been demonstrated to regulate MAPK activity. DsPTP1 (At3g23610) dephosphorylates MPK4 [52], and MAPK  PHOSPHATASE2 (MKP2; At3g06110) dephosphorylates MPK3 and MPK6 [54].

Although a MAPK regulated by IBR5 has not been reported, MAPK signaling has been implicated in both auxin and ABA responses, and both of these pathways are defective in *ibr5*. For example, transient expression in protoplasts of constitutively active MAPK kinase kinase protein ANP1 or the tobacco homolog NPK1 results in decreased auxin-responsive transcription and activation of AtMPK3 and AtMPK6 [55,56], the targets of the MKP2 phosphatase [54]. Additionally, auxin treatment activates a ~44-kD MAPK in Arabidopsis [57]. The failure of IBR5^C129S ^to fully restore *ibr5-1 *mutant phenotypes indicates that IBR5 phosphatase activity is required for full auxin and ABA responsiveness, and it will be interesting to learn if any of the MAP kinases implicated in hormone responsiveness are IBR5 substrates. The partial rescue of *ibr5 *defects observed when IBR5^C129S ^was overexpressed might result from the IBR5^C129S ^protein binding to and thus sequestering IBR5 substrate(s), as has been suggested for overexpression of the catalytically inactive MAPK phosphatase Pyp1^C470S^, which results in a phenotype similar to a loss-of-function allele of the substrate MAPK Spc1 in *Schizosaccharomyces pombe *[58,59].

In an effort to clarify the pathways through which IBR5 affects auxin responses, we examined genetic interactions between *ibr5 *and the auxin-response mutants *tir1*, *axr1*, and *aux1*. The TIR1 F-box protein acts with Aux/IAA proteins as an auxin receptor [19,20,22]. *tir1 *appeared to enhance all *ibr5 *auxin-related physiological phenotypes examined, including response defects to applied natural and synthetic auxins (Figure 1C, D) and auxin transport inhibitors (data not shown). In addition, the *tir1 ibr5 *mutant displayed a longer primary root (Figures 1C, D) and fewer lateral roots than either single mutant on unsupplemented medium (Figure 2A), suggesting that the double mutant has enhanced resistance to endogenous auxin as well.

In addition to influencing auxin responses, IBR5 modulates certain ABA responses [37]. We found not only that *tir1 *enhanced *ibr5 *ABA resistance, but also that *tir1 *itself exhibited substantial ABA resistance in the root elongation assay (Figure 2C). Although *tir1 *has not previously been reported to be ABA resistant, it has been characterized as glucose resistant [60], a phenotype common to many ABA-resistant mutants (reviewed in [61,62]). Because *tir1 ibr5 *displays enhanced resistance to both auxin and ABA, and because the *ibr5-1 *allele is a likely null [37], these results are consistent with the possibility that TIR1 and IBR5 promote auxin and ABA responsiveness independently of one another.

AXR1 is a subunit of the RUB-activating enzyme involved in RUB modification of CULLIN proteins. CULLIN is the backbone for the over 600 putative SCF complexes in Arabidopsis [63], including SCF^TIR1 ^[64]; therefore, *axr1 *mutants have pleiotropic phenotypes, including auxin resistance. The only examined phenotypes that appeared to be additive in *axr1 ibr5 *were the long primary root on unsupplemented media and aberrant vascular development (Figure 3). Unlike *ibr5*, *tir1-1 *enhances the root elongation resistance of *axr1-12 *to 2,4-D [38]. The pleiotropic nature of the *axr1 *defect and the extreme auxin resistance of the *axr1 *single mutant complicate the interpretation of our results. Regardless, it is interesting that *axr1 *and *ibr5 *mutants, which are both less sensitive to the inhibitory effects of auxin on root elongation, seem to have opposite effects on AXR3NT-GUS stability (Figure 6A; [16]).

We also examined double mutants of *ibr5 *with *aux1*, a mutant defective in an auxin influx carrier [32,34,35]. The shoots of the *aux1 ibr5 *mutant resembled *ibr5*, whereas the roots of *aux1 ibr5 *were most similar to *aux1*. Because *aux1 *lacks shoot phenotypes and most *aux1 *root phenotypes are more dramatic than *ibr5 *root defects, these results are consistent with results expected from additive defects, although the extreme resistance of *aux1 *to 2,4-D and ABA prevented us from determining whether *aux1 ibr5 *had additive defects in response to these hormones.

*ibr5 *has decreased DR5:GUS activity when grown on unsupplemented media [37], and in this study we found defects in auxin-induced DR5:GUS activity in particular tissues (Figure 5). However, microarray analysis of mRNA accumulation in 7-day-old *ibr5 *and wild-type seedlings did not reveal any dramatic (> 2.5-fold) alterations in transcripts represented in the analysis [37]. These results suggested that any gene expression changes in *ibr5 *might be subtle or local, as in the DR5:GUS analysis, and therefore not apparent in whole seedling RNA.

Because auxin-response transcripts are regulated by Aux/IAA protein stability, we sought to analyze Aux/IAA degradation in *ibr5*. Auxin promotes Aux/IAA protein degradation by mediating interaction of these repressors with SCF^TIR1 ^[19-21,65]. Several auxin-resistant mutants exhibit stabilized Aux/IAA proteins or Aux/IAA reporters, including mutants defective in the auxin receptors TIR1 [16] and the related AFB1, AFB2, and AFB3 F-box proteins [21]; other SCF components such as CUL1 [41]; and proteins that modify SCF activity, such as AXR1 [16], ECR1 [28], ETA3/SGT1b [43], and ETA2/CAND1 [42]. This stabilization probably accounts for the reduced levels of auxin-responsive transcripts reported in many of these mutants [21,25,42,45,46,66]. Like previously characterized auxin-response mutants, *ibr5 *exhibits reduced accumulation of the auxin-responsive *DR5:GUS *reporter. Unlike these other mutants, however, AXR3NT-GUS activity was not increased in *ibr5*. Moreover, IAA28myc protein was less abundant in *ibr5*, which is not expected if Aux/IAA proteins are generally stabilized, also suggesting that IBR5 modulates auxin-responsive transcription without stabilizing Aux/IAA proteins. Of course, there are 29 *Arabidopsis *members of the Aux/IAA family [7], and it remains possible that *ibr5 *specifically stabilizes certain Aux/IAA family members to reduce transcriptional activation without affecting AXR3/IAA17 or IAA28 stability.

Regardless of whether Aux/IAA proteins that remain to be assessed turn out to be stabilized in *ibr5*, *ibr5 *is the only examined auxin-response mutant [16,21,28,41-44] that does not exhibit AXR3NT-GUS stabilization. This phenotypic bifurcation may be useful in dissecting the roles of additional auxin-response mutants. Recent experiments suggest that the MYB77 transcription factor promotes auxin responses and can dimerize with ARF proteins [67]. It will be interesting to learn whether Aux/IAA proteins are stabilized in the *myb77 *mutant and whether IBR5 regulates this or some other factor needed to promote auxin-responsive transcription once the Aux/IAA repressors have been degraded. One of many possible scenarios is that IBR5 normally dephosphorylates and inactivates a MAPK that negatively regulates a transcription factor needed for auxin responses, providing a mechanism to fine-tune auxin responses without modulating Aux/IAA stability.

## Conclusion

IBR5 resembles dual-specificity phosphatases, and in this work we provide evidence that IBR5 phosphatase activity is necessary for full auxin and ABA responsiveness. Analysis of double mutants between *ibr5-1 *and several other auxin-response mutants revealed that IBR5 appears to affect auxin responses independently of the TIR1 auxin receptor. Because transcriptional repression of auxin-responsive genes is relieved by Aux/IAA protein degradation, we examined the stability of two Aux/IAA reporters in *ibr5 *and found that these proteins were not stabilized in *ibr5*, suggesting that IBR5 acts downstream of auxin recognition by the SCF^TIR1/AFB^-Aux/IAA complexes. Future determination of IBR5 substrates may allow a more detailed understanding of how this apparent dual-specificity phosphatase is able to promote auxin responses without destabilizing Aux/IAA proteins.

## Methods

### Plant materials and growth conditions

*Arabidopsis thaliana *accession Colombia (Col-0) was the wild type used for all experiments. The *ibr5-1 *mutant contains a nonsense mutation at IBR5 amino acid 42, resulting in a truncated product lacking the catalytic domain [37]. The *aux1-7 *mutant contains a missense mutation resulting in glycine 459 being replaced by aspartic acid [32]. The *tir1-1 *mutant contains a missense mutation resulting in glycine 147 being replaced by an aspartic acid [38]. The *axr1-3 *mutant contains a missense mutation resulting in cysteine 154 being replaced by a tyrosine [26].

Surface-sterilized [68] seeds were plated on PNS (plant nutrient medium with 0.5% [w/v] sucrose) [69] solidified with 0.6% (w/v) agar. Hormones used were from 0.1-, 1.0-, or 100-mM stocks in ethanol, with ethanol-supplemented media used as controls, and all treatments normalized to the same ethanol content (less than 0.1 μL ethanol/mL medium). Seedlings were grown at 22°C under continuous light. Unless indicated otherwise, plates were incubated under yellow long-pass filters to slow the breakdown of indolic compounds [70]. Plants were grown in soil (Metromix 200; Scotts, Marysville, OH) at 22 to 25°C under continuous illumination by cool-white fluorescent bulbs (Sylvania, Danvers, MA).

### IBR5^C129S ^construct

The pKS*IBR5*c construct [37] was mutated using oligonucleotide-directed mutagenesis [71] to alter the presumptive catalytic cysteine at amino acid position 129 to a serine using a primer 5'-CTTTCCCAGACATCGAATGCACAAGAAC-3' (altered residues underlined) designed to contain a *Taq*1α restriction site. The mutant cDNA was then excised using *Not*I and subcloned into the plant transformation vector 35SpBARN [72] between the Cauliflower mosaic virus 35S promoter and the *nos *terminator. The 35S:*IBR5*^C129S ^plasmid was electroporated [71] into *Agrobacterium tumefaciens *GV3101, which was used to transform wild-type Col-0 and *ibr5-1*. Transformants were identified on PNS medium supplemented with 10 μg/mL BASTA (glufosinate-ammonium) after 10 days under white light. Homozygous lines were identified in subsequent generations by examining the pattern of BASTA resistance.

### *IAA28myc *construct

The *IAA28:IAA28myc *construct [73] contains the *IAA28 *genomic region, including 3.1 kb of DNA 5' of the *IAA28 *coding sequence, subcloned into the pBIN19 plant transformation vector. The last exon of *IAA28 *has been modified in this construct to encode five copies of the c-Myc epitope immediately upstream of the *IAA28 *termination codon. The *IAA28:IAA28myc *plasmid was electroporated [71] into *Agrobacterium tumefaciens *GV3101, which was used to transform wild-type Col-0. Transformed seedlings were identified on PN medium supplemented with 12 μg/ml kanamycin after growth under white light. Homozygous single-insert lines were identified by examining the pattern of kanamycin resistance in subsequent generations. *ibr5-1*, *tir1-1*, and *axr1-3 *were crossed to a wild-type (Col-0) single-insert line to generate *ibr5*, *tir1*, and *axr1 *carrying the *IAA28:IAA28myc *construct.

### Phenotypic analyses

All assays were conducted at least three times with similar results. For auxin-response root-elongation assays, seedlings were grown for 8 or 9 days on PNS with the indicated auxin concentrations and removed from the agar, and the lengths of the primary roots were measured. For ABA response root-elongation assays, seedlings were grown for 4 days on PNS to allow efficient germination, then were transferred to PNS supplemented with either ethanol or ABA. After an additional 4 days of growth, primary root lengths were measured.

In lateral root assays, seedlings were grown for 4 days on PNS, transferred to PNS supplemented with either ethanol or 10 μM IBA, and grown for an additional 4 days, after which lateral roots were examined under a dissecting microscope. Primordia emerged from the primary root were counted as lateral roots.

For hypocotyl elongation assays, seeds were plated on media supplemented with either ethanol or 20 μM IBA. After 1 day in the light, plates were wrapped with aluminum foil and incubated for an additional 4 days in the dark, after which seedlings were removed from the agar and hypocotyls lengths were measured.

To examine cotyledon vascular patterns, seedlings were grown for 8 days on PNS. Chlorophyll was removed using an ethanol series, and seedlings were cleared by incubating for one week at room temperature in chloral hydrate solution (80 g chloral hydrate, 20 mL glycerol, and 10 mL water). Cleared seedlings were mounted and photographed through a dissecting microscope.

### Double mutant isolation

The *ibr5-1 *mutant was crossed to *aux1-7 *[39], *axr1-3 *[30], and *tir1-1 *[38], all in the Col-0 accession. Double mutants were identified by PCR analysis of F2 plants. Amplification of *AUX1 *with AUX1-3 (5'-CATGGGTCAACAAAGCTTTGGATTTTGTCC-3') and AUX1-4 (5'-TTCGTGACTTTTACTCCCTTCACGTATACG-3') yields a 464-bp product with two *Dpn*II restriction sites in wild type and three in *aux1-7*. Amplification of *AXR1 *with the derived cleaved amplified polymorphic sequence primer [74,75] AXR1-*Acc*1 (5'-AAACCAACTTAACGTTTGCATGTCG-3'; altered residue underlined) and AXR1-15 (5'-TCTCATATGTACTTTTCCTCGTCCTCTTCAC-3') yields a 185-bp product with one *Acc*1 restriction site in wild type and none in *axr1-3*. Amplification of *TIR1 *with TIR1-3 (5'-TTGAAGAGATAAGGCTGAAGAGGATGG-3') and TIR1-4 (5'-TACACCACCGTTAAATAAGACCCACCAGAAAG-3') yields a 488-bp product with one *Dpn*II restriction site in wild type and two restriction sites in *tir1-1*. PCR-based identification of *ibr5-1 *was as described previously [37].

### Northern analysis

Surface-sterilized [68] seeds were plated on filter paper-lined PNS and grown under continuous illumination. After 6 days, the filter paper with seedlings was lifted off the agar surface. Seedlings were floated in liquid PN supplemented with mock (ethanol) or 200 nM IAA for 10 minutes. Seedlings were collected and ground with a mortar and pestle in liquid nitrogen. RNA was extracted using TriReagent (Sigma) according to the manufacturer's instructions. Total RNA (10 μg) was electrophoresed on a 1% agarose gel containing 0.37 M formaldehyde [71] and transferred to a positively-charged nylon 66 membrane (Roche Applied Science, Indianapolis, IN) in 20× SSC using capillary action. DIG-labeled probe was hybridized in DIG Easy Hyb buffer (Roche) overnight at 50°C and washed at moderate stringency according to the manufacturer's instructions. Probes were detected using an alkaline phosphatase-conjugated anti-DIG antibody (Roche) diluted 1:20,000 in Blocking Buffer (Roche), then visualized using a 1:200 dilution of CDP-Star (Roche).

DIG-labeled *IAA28 *probe was synthesized by PCR amplification using a PCR DIG Probe Synthesis Kit (Roche) according to the manufacturer's instructions from a cDNA template [48] using T1N24-20 (5'-CCATCGAACTGATGATTTTGGCC-3') and T1N24-21 (5'-CCTCCTTGTCACCAATTCACTTCC-3'), yielding a 525-bp product.

### Immunoblot analysis

To visualize IBR5, protein was extracted from entire 2-day-old seedlings grown in 0.1% agar under white light by grinding frozen tissue with a pestle and adding one volume NuPAGE 2X LDS buffer (Invitrogen, Carlsbad, CA). Debris was pelleted by centrifugation for 4 minutes. The supernatant was heated to 100°C for 5 minutes. Protein extracts were separated by SDS-polyacrylamide gel electrophoresis beside Cruz markers (Santa Cruz Biotechnology, Santa Cruz, CA) using a NuPAGE 10% Bis-Tris gel and MES running buffer (Invitrogen).

Protein was transferred for 35 min at 24 V to a Hybond ECL nitrocellulose membrane (Amersham Pharmacia Biotech, Piscataway, NJ) using NuPAGE transfer buffer (Invitrogen). After blocking for 1 hour in 8% powdered milk in Tween Tris-buffered saline (TTBS; [71]), the membrane was incubated overnight at 4°C with affinity purified IBR5 antibody [37] diluted 1:20 in blocking buffer. The membrane was then washed three times with TTBS and incubated with HRP-conjugated goat anti-rabbit secondary antibody (Santa Cruz Biotechnology) diluted 1:2000 for 1 hour, washed again as described before, then visualized using LumiGLO reagent (Cell Signaling, Beverly, MA). Membranes subsequently were incubated with an antibody against spinach (*Spinacia oleracea*) HSC70 (Stressgen Bioreagents SPA-817) diluted 1:5000, followed by HRP-conjugated goat anti-mouse secondary antibody (Santa Cruz Biotechnology) diluted 1:2000, and visualized using LumiGLO reagent (Cell Signaling).

To visualize IAA28myc, 10-day-old light-grown seedlings were removed from PNS medium and floated in liquid PN supplemented with either ethanol (mock) or 200 nM IAA. At the indicated time points, roots were excised and protein was extracted. For MG132 treatment, 3-day-old light-grown seedlings were removed from PNS medium and suspended in water containing either DMSO (mock) or 300 μM MG132. After 1 or 2 hours, protein was extracted from whole seedlings. In both experiments, protein was separated by SDS-polyacrylamide gel electrophoresis and transferred to a membrane as described above. After blocking for 1 hour in 8% powdered milk in TTBS, the membrane was incubated overnight at 4°C with monoclonal 9E10 anti-c-Myc antibody (Santa Cruz Biotechnology SC-40) diluted 1:500 and anti-HSC70 antibody (Stressgen Bioreagents SPA-817) diluted 1:250,000 in blocking buffer. The membrane was then washed three times with TTBS and incubated with HRP-conjugated goat anti-mouse secondary antibody (Santa Cruz Biotechnology) diluted 1:2000 for 4 hours, washed again as described before, then visualized using LumiGLO reagent (Cell Signaling).

### HS:AXR3NT-GUS analysis

Wild-type (Col-0) lines carrying *HS:AXR3NT-GUS *and *HS:axr3-1NT-GUS *and *axr1-3 *carrying *HS:AXR3NT-GUS *were described previously [16]. To obtain additional lines carrying these reporters, *ibr5-1 *was crossed to wild type carrying *HS:AXR3NT-GUS *and to wild type carrying *HS:axr3-1NT-GUS*. *tir1 ibr5 *was crossed to wild type carrying *HS:AXR3NT-GUS *to obtain *tir1-1 HS:AXR3NT-GUS *and *tir1 ibr5 HS:AXR3NT-GUS*. Mutants were identified by PCR analysis of the F2 plants, as described above. The presence of *HS:AXR3NT-GUS *or *HS:axr3-1NT-GUS *was assayed by resistance to kanamycin and confirmed by GUS staining.

For histochemical assays, 8-day-old light-grown seedlings were removed from PNS plates and floated in 0.5 mL liquid PN medium diluted six-fold with water (for hormone response assays) or sterile water (for MG132 assays) contained in a 12-well plate. Two milliliters of prewarmed (37°C) one-sixth liquid PN or prewarmed water was added to each well, and the plates were incubated at 37°C for 2 hours. For the hormone response assays, seedlings were transferred to room temperature for 20 minutes prior to 10- and 20-minute mock (ethanol) or 100 nM IAA treatments, or 40-minute mock (ethanol) or 10 μM IBA treatment. For MG132 assays, seedlings were incubated in the dark, and either DMSO (mock) or MG132 (to 50 μM) was added to each well 1 hour into the heat treatment. After heat treatment, plates were transferred to room temperature for 2 hours. Seedlings were stained for GUS activity as previously described [76].

## Authors' contributions

LCS isolated and characterized double mutants, analyzed transcripts, performed GUS experiments, IAA28 western analyses, and characterized the phenotypes of *35S:IBR5*^*C*129*S *^lines. MMA made *35S:IBR5*^*C*129*S *^constructs, transformed them into plants, and performed IBR5 western analyses. BB conceived and coordinated the study. All authors participated in drafting and editing the manuscript, and read and approved the final manuscript.

## Supplementary Material

Additional file 1 Normalized *ibr5 *double mutant auxin response defects. The auxin responses of *ibr5 *and double mutants are represented after normalization to root lengths of mock-treated seedlings. Click here for file

Additional file 2 Normalized auxin-response mutant defects in lateral root induction by IBA, hypocotyl elongation inhibition by IBA, and root elongation inhibition by ABA. The IBA and ABA responses of *ibr5 *and double mutants are represented after normalization to mock-treated seedlings. Click here for file
