# Supplementary material for: The IBR5 phosphatase promotes Arabidopsis auxin responses through a novel mechanism distinct from TIR1-mediated repressor degradation
Source: BMC Plant Biol. 2008 Apr 18;8:41. doi: 10.1186/1471-2229-8-41 (PMC2374786; doi:10.1186/1471-2229-8-41)
Supplement: Additional file 1 — Normalized ibr5 double mutant auxin response defects. The auxin responses of ibr5 and double mutants are represented after normalization to root lengths of mock-treated seedlings. [file 1471-2229-8-41-S1.pdf]

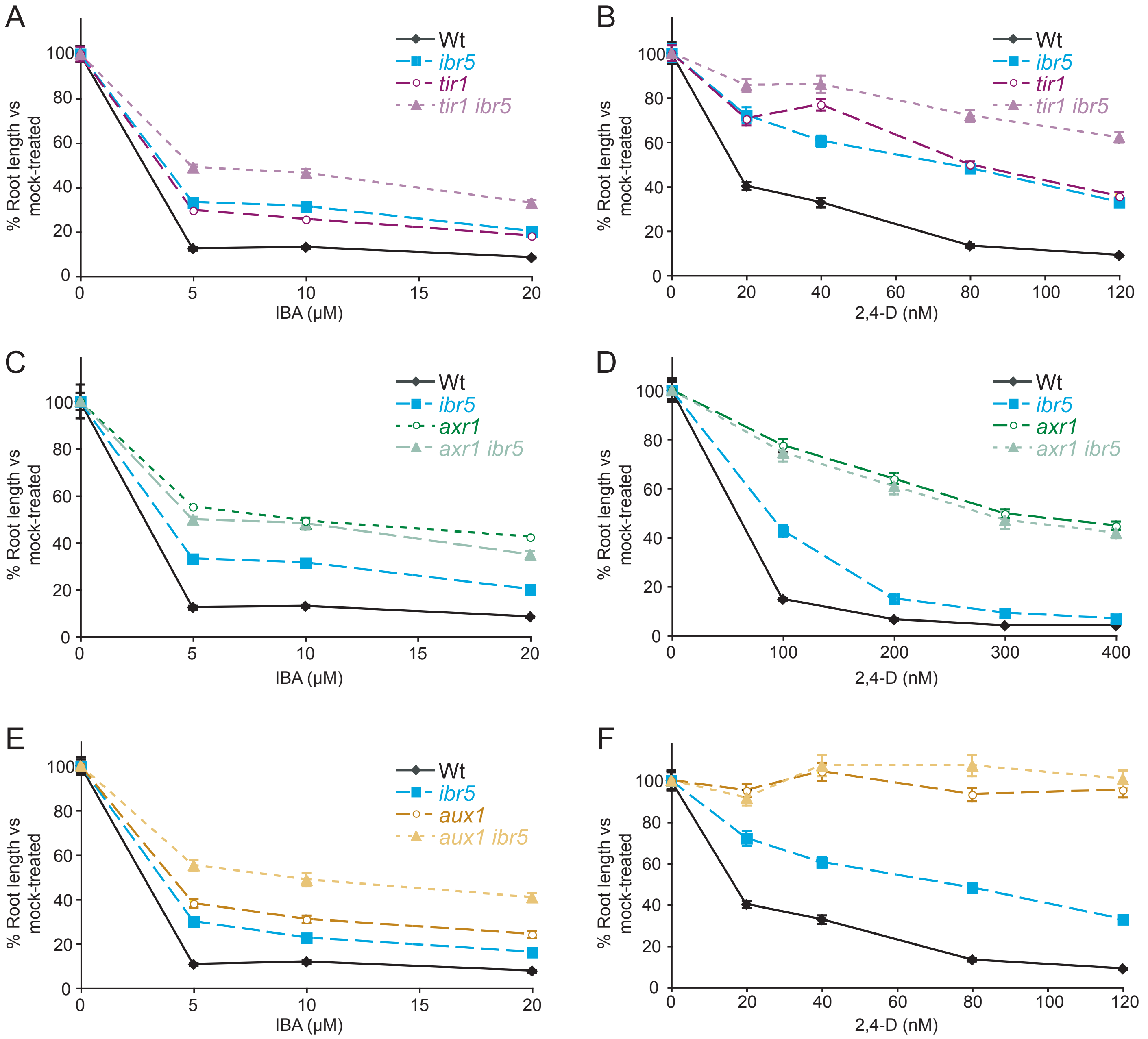

Additional File 1 - Normalized *ibr5* double mutant auxin response defects

Normalized primary root lengths of 8-day-old (A, B, D, E, F) or 9-day-old (C) Col-0 (Wt), *ibr5-1*, *tir1-1*, *tir1-1 ibr5-1*, *axr1-3*, *axr1-3 ibr5-1*, *aux1-7*, and *aux1-7 ibr5-1* seedlings grown under yellow-filtered light at 22 °C on medium supplemented with various concentrations of IBA (A, C, E) or 2,4-D (B, D, F). Root elongation data were normalized by comparing all root length values to the mean root length on mock-treated media and are presented as the percent root length of treated versus mock-treated. Panels A and B correspond to Figures 1C and 1D, respectively, from the main text. Panels C and D correspond to Figures 3C and 3D. Panels E and F correspond to Figures 4C and 4D. (A, B) *ibr5-1* enhances *tir1-1* auxin-response defects on IBA (A) and 2,4-D (B). Error bars represent standard errors of the means (*n* ≥ 18). (C, D) *ibr5-1* does not enhance *axr3-1* auxin-response defects on IBA (C) or 2,4-D (D). Error bars represent standard errors of the means (*n* ≥ 15). (E, F) *ibr5-1* enhances *aux1-7* auxin-response defects on IBA (E) but not on 2,4-D (F). Error bars represent standard errors of the means (*n* ≥ 16).
