# Supplementary material for: The IBR5 phosphatase promotes Arabidopsis auxin responses through a novel mechanism distinct from TIR1-mediated repressor degradation
Source: BMC Plant Biol. 2008 Apr 18;8:41. doi: 10.1186/1471-2229-8-41 (PMC2374786; doi:10.1186/1471-2229-8-41)
Supplement: Additional file 2 — Normalized auxin-response mutant defects in lateral root induction by IBA, hypocotyl elongation inhibition by IBA, and root elongation inhibition by ABA. The IBA and ABA responses of ibr5 and double mutants are represented after normalization to mock-treated seedlings. [file 1471-2229-8-41-S2.pdf]

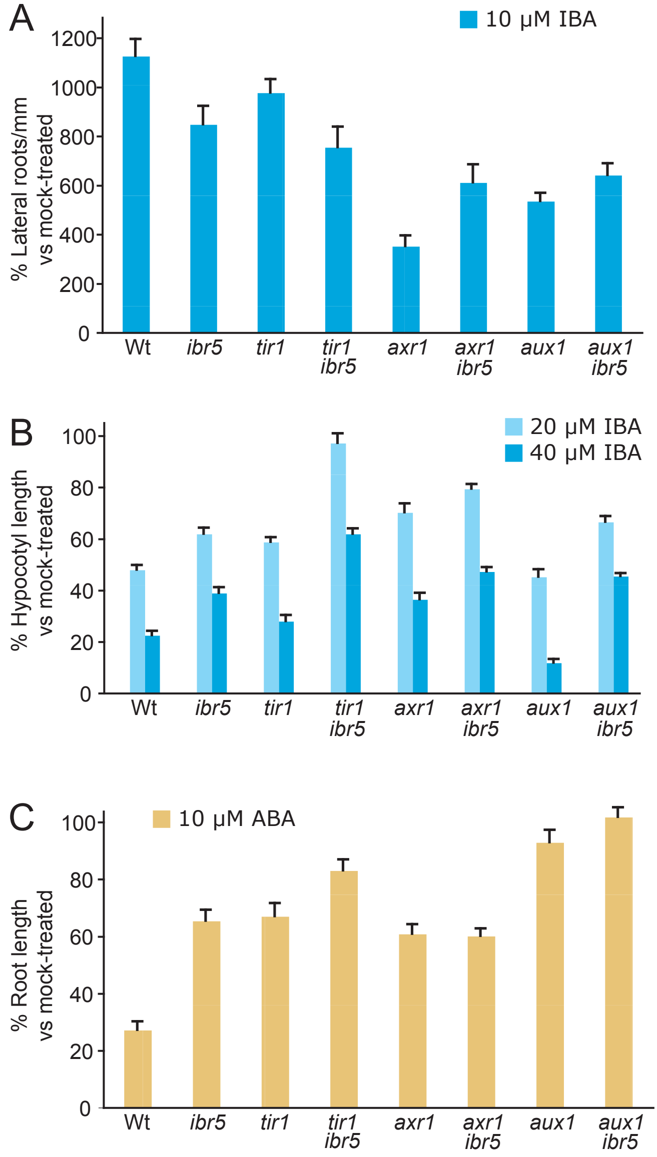

Additional File 2 - Normalized auxin-response mutant defects in lateral root induction by IBA, hypocotyl elongation inhibition by IBA, and root elongation inhibition by ABA

Hormone response of Col-0 (Wt), *ibr5-1*, *tir1-1*, *tir1-1 ibr5-1*, *axr1-3*, *axr1-3 ibr5-1*, *aux1-7*, and *aux1-7 ibr5-1* were examined. Data from Figure 2 in the main text were normalized by comparing each individual to the mean mock-treatment value and are presented as the percent of mock-treated. (A) Lateral roots were counted 4 days after transfer of 4-day-old seedlings to medium supplemented with either 0 (ethanol control) or 10 μM IBA. Primordia emerged from the main root were counted as lateral roots. Error bars represent standard errors of the means ($n \geq 14$). (B) Hypocotyl lengths were measured 4 days after transfer of 1-day-old seedlings to the dark. Error bars represent standard errors of the means ($n = 20$). (C) Lengths of primary roots 4 days after transfer of 4-day-old seedlings to medium supplemented with either 0 (ethanol control) or 10 μM ABA. Error bars represent standard errors of the means ($n \geq 14$).
